# Supplementary material for: Association between oral microbiome and seven types of cancers in East Asian population: a two-sample Mendelian randomization analysis
Source: Front Mol Biosci. 2023 Nov 21;10:1327893. doi: 10.3389/fmolb.2023.1327893 (PMC10702768; doi:10.3389/fmolb.2023.1327893)
Supplement: Supplementary file 1 [file DataSheet1.ZIP › Supplementary Materials/MR plots of saliva/saliva═╝/colorectal cancer/pheno.2045_to_colorectal cancer_forest.pdf]

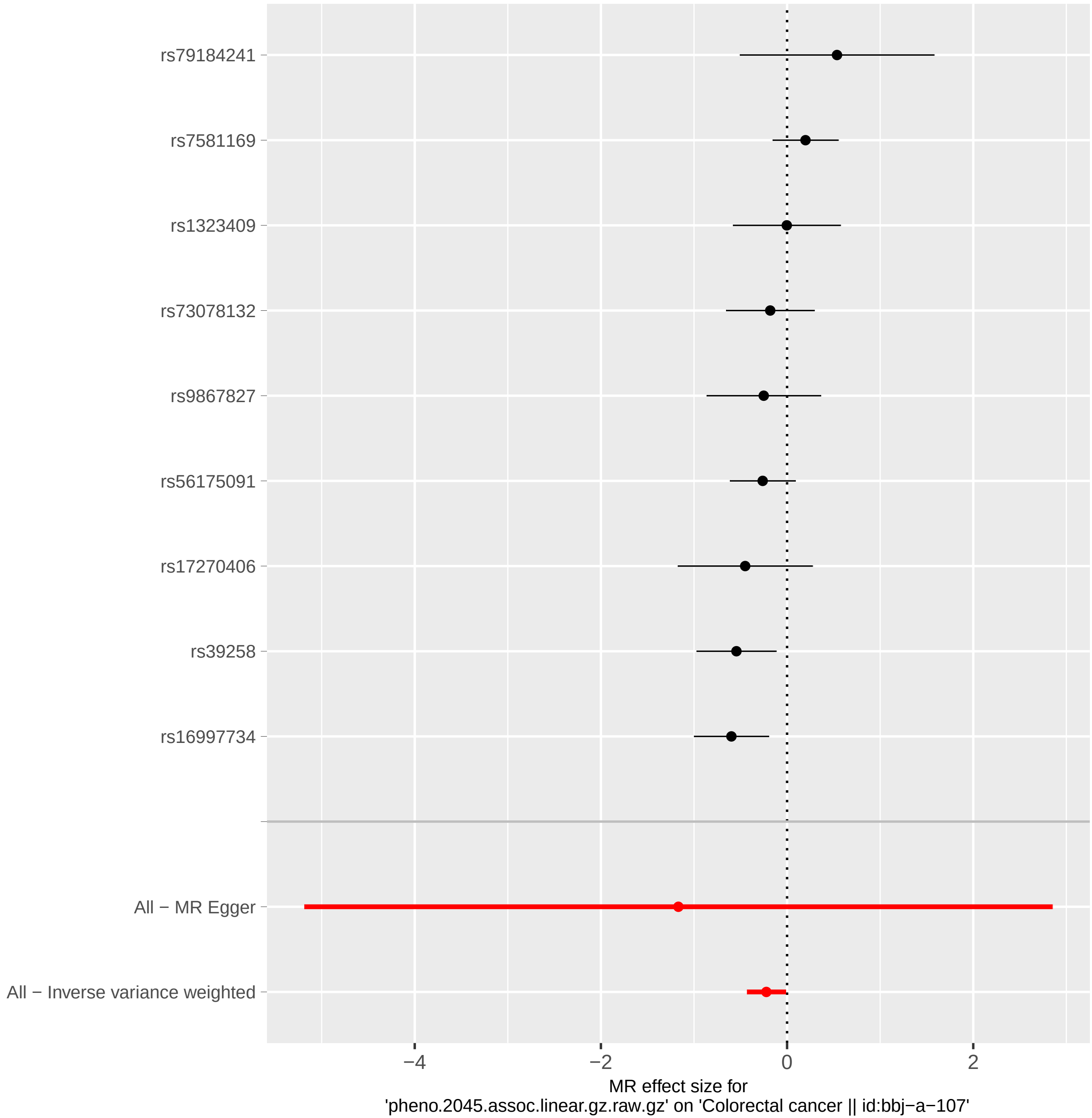

rs79184241
rs7581169
rs1323409
rs73078132
rs9867827
rs56175091
rs17270406
rs39258
rs16997734
All − MR Egger
All − Inverse variance weighted
−4
−2
0
2
MR effect size for
'pheno.2045.assoc.linear.gz.raw.gz' on 'Colorectal cancer || id:bbj−a−107'
